# Toward Therapeutic Targeting of Bone Marrow Leukemic Niche Protective Signals in B-Cell Acute Lymphoblastic Leukemia

**DOI:** 10.3389/fonc.2020.606540

**Published:** 2021-01-08

**Authors:** Marjorie C. Delahaye, Kaoutar-Insaf Salem, Jeoffrey Pelletier, Michel Aurrand-Lions, Stéphane J. C. Mancini

**Affiliations:** Aix Marseille University, CNRS, INSERM, Institut Paoli-Calmettes, CRCM, Marseille, France

**Keywords:** B-cell acute lymphoblastic leukemia, bone marrow, leukemic niches, targeted treatments, B cell development

## Abstract

B-cell acute lymphoblastic leukemia (B-ALL) represents the malignant counterpart of bone marrow (BM) differentiating B cells and occurs most frequently in children. While new combinations of chemotherapeutic agents have dramatically improved the prognosis for young patients, disease outcome remains poor after relapse or in adult patients. This is likely due to heterogeneity of B-ALL response to treatment which relies not only on intrinsic properties of leukemic cells, but also on extrinsic protective cues transmitted by the tumor cell microenvironment. Alternatively, leukemic cells have the capacity to shape their microenvironment towards their needs. Most knowledge on the role of protective niches has emerged from the identification of mesenchymal and endothelial cells controlling hematopoietic stem cell self-renewal or B cell differentiation. In this review, we discuss the current knowledge about B-ALL protective niches and the development of therapies targeting the crosstalk between leukemic cells and their microenvironment.

## Introduction

B cell Acute Lymphoblastic Leukemia (B-ALL) is characterized by differentiation arrest and malignant transformation of developing bone marrow (BM) B cells. B-ALL is the most common cancer during childhood and is relatively well treated, with a 5-year overall survival reaching 90% (1). The treatment is largely based on chemotherapy and despite the good prognosis, 20% of children relapse with a survival rate of 30% (2). The incidence of B-ALL for adults is lower than for children but concerns a similar number of patients. While adult patient management has also improved and allowed the mortality rate to decrease, prognosis remains poor with 60% of relapse and a median survival of 3 to 6 months following salvage chemotherapies (3–5). Furthermore, there is a high risk to develop long term sequelae related to chemotherapy, including secondary neoplasms, chronic health conditions, or endocrine dysfunctions (6).

Leukemia transformation is driven by a combination of genetic abnormalities including translocations, MLL rearrangements, hyper and hypodiploid karyotypes, gene deletions, point mutations, which lead to aberrant expression of constitutively active or inactive regulatory proteins (7). In addition to cell-autonomous alterations of key regulatory pathways, growing evidences support the influence of non-cell autonomous cues on tumor progression and resistance to therapies that are transmitted by cells of the BM microenvironment (8). These supportive cellular niches are composed of endothelial, immune, and mesenchymal cells [including C-X-C Motif Chemokine Ligand 12 (CXCL12) abundant reticular (CAR) cells, osteolineage cells, adipocytes] which have the capacity to secrete soluble factors and to establish direct contacts with leukemic cells. Adrenergic nerves were also found to regulate early hematopoiesis as the β2 and β3 adrenergic receptors expressed by the microenvironment are oppositely involved in myeloid and lymphoid skewing of hematopoietic stem cells (HSC) respectively (9, 10). Finally, non-cell autonomous cues related to the hypoxic nature of the BM also play an important role in lymphoid development. However, despite tremendous progress made in the cellular and molecular characterization of normal differentiating B cell niches (11), the knowledge on B-ALL supportive niches remains relatively limited. Therefore, because of the influence of the microenvironment on leukemic growth and chemoresistance, targeting the leukemic niche could represent an attractive therapeutic adjuvant therapy to improve current treatments.

In this review, we present how the BM microenvironment can influence development and survival of normal and leukemic B cells. We also discuss the potential of new innovative therapeutic strategies targeting the crosstalk between leukemic cells and their microenvironment.

## Normal B Cell Niches

The organization of the different steps of B lymphopoiesis in the BM is conserved between mouse and human and allows the acquisition of a diverse repertoire of non-autoreactive B cell receptors (BCR). B cell commitment is definitive upon B cell progenitor (pre-pro-B cell) entry in the pro-B cell stage and Pax5 upregulation (12). In mouse and human, pre-pro-B cells are marked by the early expression of pre-BCR and BCR components (CD79a, VpreB) (13, 14). Upon Pax5 expression, pro-B cells upregulate CD19, RAG (recombination activating gene) proteins, and the terminal deoxynucleotidyl transferase (TdT) involved in immunoglobulin heavy chain (IgH) gene recombination. As recombination is a random process forming imprecise junctions between variable gene segments coding for the IgH chain, an initial step of interleukin-7 (IL7)-dependent proliferation increases the odds to express a functional IgH chain and thus increases diversity (15). The IgH chain is then expressed at the pre-B cell stage as part of a pre-BCR, following association with the surrogate light chain (SLC), composed of the invariant λ5 (λ-like in human) and VpreB proteins, and with the CD79a/CD79b signaling complex. Again to increase diversity, Pre-BCR signaling induces clonal expansion of pre-B cells before the initiation of rearrangements of genes coding for the Ig light chain (IgL) in late pre-B cells. Finally, functional IgL chains are associated to the IgH chain at the immature B cell stage to form the BCR. Immature B cells expressing a non-autoreactive BCR can leave the BM to finish their maturation.

Different cellular niches control these differentiation steps through secretion of growth factors and direct cell-cell interactions. Among soluble factors, CXCL12 and IL7 are essential for early B cell development. The main function of CXCL12 is to retain early B cells in the BM, close to their nurturing niches, although a role on lymphopoiesis cannot be excluded as it synergizes with IL7 to induce early B cell proliferation and survival *in vitro* (16–19). In addition to its role as a chemoattractant, CXCL12 induces adhesion of pro-B and pre-B cells by activating the interaction of α4β1-integrin (VLA-4) to VCAM-1 through a focal adhesion kinase (FAK)-dependent pathway (20, 21). VLA-4 expression was indeed shown to be crucial for early B cell development in VLA4 deficient mice (22). Importantly, the decreased expression of CXCR4, receptor for CXCL12, favors immature B cells egress to the periphery through downregulation of VCAM-1-mediated adhesion (23).

In mouse, IL7 is responsible for pre-pro-B and early pro-B cell proliferation (24). In human, as B cells were still found in the peripheral blood of patients presenting defects in IL7 receptor (IL7R) signaling, it was first proposed that IL7 was not required for B cell development (25). However, recent results have demonstrated that IL7 induces human B cell development (26–28). This discrepancy is probably due to the presence of IL7-independent B cells originating from fetal life in patients compromised for IL7R signaling, similarly to what was observed for IL7^−/−^ mice (29).

**Figure 1:**
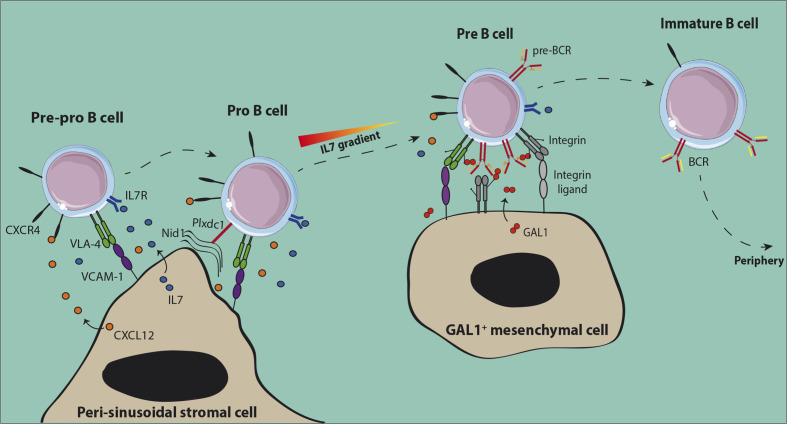
Normal early B cell niches in the bone marrow. Both pre-pro-B and pro-B cells are in contact with peri-sinusoidal stromal cells which express high levels of CXCL12 and IL-7. Upon expression of the pre-BCR, pre-B cells move to IL-7^low^ regions close to Galectin-1 (GAL1) expressing stromal cells. GAL1-dependent cross-linking of the pre-BCR induces proliferation of pre-B cells before the initiation of IgL rearrangements. After expression of the BCR, non-autoreactive immature B cells leave the BM to achieve their maturation in the periphery.

BM niches for early differentiating B cells have been identified recently (**Figure 1**). The presence of CXCL12 expressing stromal cells associated to BM sinusoids was demonstrated using mice with a knock-in of Green Fluorescent Protein (GFP) under the control of the Cxcl12 promoter (30, 31). Pre-pro-B cells were found in contact with these peri-sinusoidal stromal (PSS) cells (**Figure 1**) (30). Later on, PSS cells were found to co-express IL7 and to support the development of pro-B cells (32–34). Importantly, human pro-B cells identified based on TdT expression were also found to be located close to similar peri-vascular stromal cells expressing both CXCL12 and IL7 (34). The specific interactions of pro-B cells with their supportive niche were further deciphered through the analysis of the trans-interactome between the partner cells and led to the identification of the ligand-receptor pair *Plxdc1/Nid1* (34). Plxdc1 is an adhesion molecule regulated by Pax5 (35) and specifically expressed by pro-B cells as compared to other B cell subsets, while Nidogen-1 is part of the extra-cellular matrix secreted by PSS cells. The analysis of *Nid1* ^−/−^ mice showed that this interaction was important for the retention in the supportive niche and consequently for the IL7-dependent proliferation of early pro-B cells. This result suggests that CXCL12/CXCR4 dependent chemo-attraction and VLA-4/VCAM-1-dependent firm adhesion of pro-B cells to PSS cells depend on initial Plxdc1/Nidogen-1 interaction.

Upon differentiation towards the pre-B cell stage, cells were shown to be located in close vicinity to Galectin-1 (GAL1) expressing mesenchymal cells which are away from the sinusoids and IL7-expressing PSS cells (32). This result is consistent with the fact that pro-B cells need high IL7 concentrations to be maintained and proliferate, while a low dose favors pre-B cell differentiation and expansion (36). GAL1 is a ligand for the pre-BCR identified in both human and mouse which binds the λ5 subunit through direct protein-protein interactions (37, 38). GAL1 is also a lectin that binds specifically β-galactosides and particularly glycosylated chains of the VLA-4, VLA-5, and LFA-1 integrins expressed by pre-B cells (39). Efficient differentiation of pre-B cells depends on specific contacts with stromal cells, involving tri-partite interactions between pre-BCR, GAL1, and integrins, and then binding of integrins with their respective ligands expressed at the surface of stromal cells (37–39).

The BM is a highly hypoxic organ with a local oxygen tension (pO_2_) ranging from 1.3% away from the endosteum to 1.8% close to it (40). Hypoxia induces a cellular response through the stabilization of the hypoxia-inducible factor (HIF)-1α and -2α and then activation of downstream effectors (41). It has been shown for human lymphoid-primed multipotent progenitors (LMPPs) that *in vitro* cultures in hypoxic versus normoxic conditions stabilize HIF-1α and HIF-2α and favor a lymphoid gene expression program (42). In mouse and human, hypoxia-dependent genes are highly expressed from pre-pro-B to pre-B cells and decreased in immature B cells (43). The B cell specific deletion of the *Vhl* (von Hippel-Lindau) gene, a E3 ubiquitin ligase which drives HIF proteins to degradation in presence of oxygen, leads to a constitutive activation of HIF proteins and a severe decrease in peripheral B cells. This phenotype was found to be HIF-1α-dependent and linked to a decrease in IgH repertoire diversity from the pro-B cell stage, a block at the immature B cell stage and a lower BCR editing. Dysregulation of HIF-1α in immature B cells leads to a decreased BCR and CD19 expression and to a higher cell death related to an increased expression of the pro-apoptotic protein BIM. Consequently, HIF-1α and more generally hypoxia contributes to the normal development of B cells.

## Leukemic B Cell Niches

For a long time, it has been considered that tumorigenesis was a cell-autonomous process. However, it has been observed 50 years ago in the case of hematopoietic cell transplantation that some patients could develop donor cell leukemia although the donor was presumably healthy and did not develop leukemia in the following months either (44). These observations were in accordance with the seed and soil theory which stands that similarly to plants, malignant cells (“seeds”) likely need a favorable or permissive microenvironment (“soil”) to grow (45).

**Figure 2:**
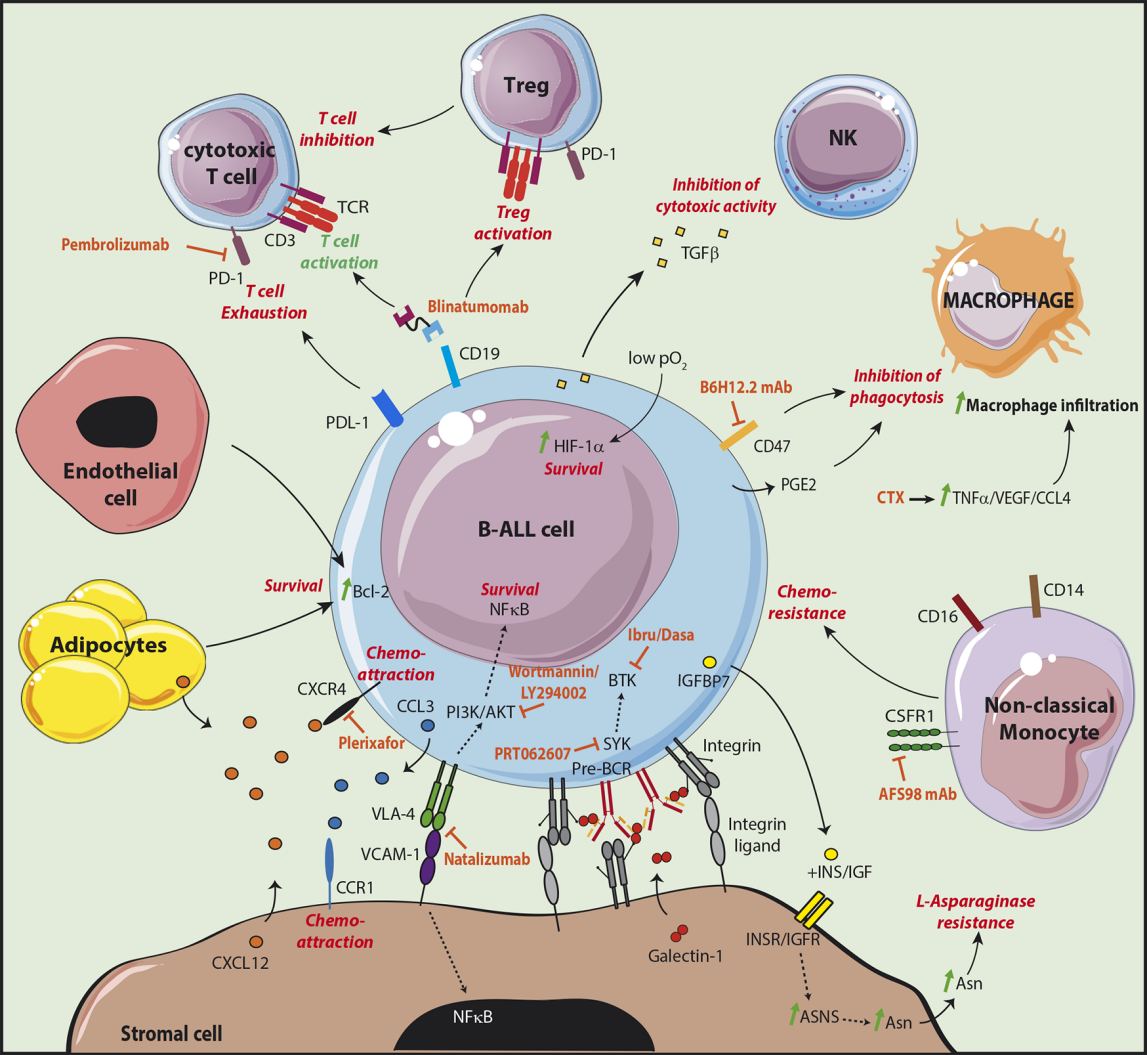
Targeting the leukemic B cell niche. The different molecular interactions of B-ALL cells with cells supporting their survival and development are shown. The supportive signals transmitted by the different partners are shown in red. The treatments targeting the supportive niche are shown in orange. The CD19/CD3 bispecific T-cell engager blinatumomab allows the redirection and the activation of cytotoxic T cells. However, an increased Treg activation was observed in non-responder patients, as well as an increase of the checkpoint molecule PD-1 on T cells and of its ligand PDL-1 on leukemic cells.

B-ALL in co-culture with stromal cells were indeed found to be protected from apoptosis, confirming that genetic alterations are not sufficient for the maintenance of leukemic cells (46). Stromal cells were further shown to protect leukemic cells from chemotherapeutic drugs *in vitro* (47). More recent studies have highlighted *in vivo* the presence of dormant cells, called leukemia initiating cells (LIC), protected by the microenvironment, which are resistant to therapy and involved in relapse (48, 49). These cells, which were identified following transplantation of primary B-ALL to immunodeficient mice, are similar to leukemic cells isolated from patients with minimal residual disease (MRD). Furthermore, they present gene expression signatures of quiescent cells and most particularly of hematopoietic stem cells (HSC). These LIC are latent sub-clones, in a dormant state, which may be rare and present at the time of diagnosis or may be the result of sub-clonal evolution (48). Their quiescence is probably induced by interactions with the microenvironment as they start proliferating and become sensitive to chemotherapy in *in vitro* cultures, similarly to bulk leukemic cells (49). The influence of stromal cells was confirmed in patient derived xenograft (PDX) mice. Upon transplantation of B-ALL cells, the invasion of BM by blast cells led to progressive damages in the vascular structure and a loss in N-Cadherin^+^ cells likely composed of stromal and osteoblastic cells (50, 51). Interestingly, following Ara-C treatment, resistant leukemic cells were found associated to stromal cells phenotypically similar to CD51^+^LepR^+^NG2^−^ PSS cells (34). Furthermore, specific expression of the chemoattractant CCL3 by the resistant leukemic cells favored migration of the CCR1^+^ protective stromal cells in their vicinity (**Figure 2**). These results confirm the existence of protective leukemic niches and that leukemic cells actively shape their own supportive niches under the pressure of chemotherapeutic treatments.

Stromal cells have the capacity to directly inhibit the effect of therapeutic agents used for B-ALL treatment. B-ALL strongly relies on extracellular sources of asparagine (Asn) related to their low expression level of Asn synthetase (ASNS) (52). Therapeutic treatments therefore take advantage of L-asparaginase to deprive leukemic cells from this crucial amino acid. Stromal cells were shown to express 20 times higher levels of ASNS than B-ALL and to be an important source of Asn probably at the origin of L-asparaginase treatment resistance (53). Interestingly, it has been observed that B-ALL in co-culture with stromal cells produce insulin-like growth factor (IGF) binding protein 7 (IGFBP7) associated with L-asparaginase resistance (54). In this study, the authors found that IGFBP7 enhanced ASNS expression and Asn secretion by stromal cells in an insulin/IGF dependent manner (**Figure 2**).

B-ALL subtypes still possess features of their normal differentiating B cell counterparts and their survival and development therefore partly rely on similar signals transmitted by stromal cell niches. B-ALL express VLA-4 and the importance of the VLA-4/VCAM-1 pair in adhesion to stromal cells was first shown *in vitro* using blocking antibodies (47, 55). Furthermore, VCAM-1 over-expression in stromal cell lines enhanced B-ALL resistance to chemotherapeutic drugs *in vitro* while VLA-4 negative variants of the B-ALL cell line Nalm6 had a decreased capacity to engraft in immunodeficient mice (56, 57). Finally, the outcome was poorer in MRD^+^ childhood B-ALL with a high expression of VLA-4 (58). Integrin mediated adhesion involves the reciprocal activation of the phosphatidylinositol-3-kinase (PI3K)/AKT signaling pathway. In B-ALL, AKT activation is responsible for the inhibition of chemotherapy-induced apoptosis and was shown to be sustained by stromal cells *in vitro* (59). In addition, the PI3K inhibitors wortmannin or LY294002 impaired the protective effect of stromal cells (56, 59). Activation of AKT was associated with a poor prognosis and to chemoresistance in childhood B-ALL (60). Finally, VLA-4/VCAM-1 interaction also induces the activation of nuclear factor (NF)-κB in both leukemic cells and stromal cells *in vitro* (61). Importantly, inhibition of the NF-kB signaling pathway in stromal cells affects their chemoprotective function *in vitro* and *in vivo*, indicating that the reciprocal activation of leukemic cells and stromal cells by integrins is a key event in chemoresistance mechanisms.

Similarly to their normal counterparts, B-ALL integrin β1-dependent adhesion to stromal cells was shown to be increased through the activation of the CXCL12/CXCR4 signaling pathway (62). However, the influence of CXCL12 as a pro-survival factor per se remains controversial (63, 64). The chemoattractive function of CXCL12 on B-ALL cell lines and patient samples was first demonstrated with transmigration assays *in vitro* (65). Furthermore, homing to the BM of immunodeficient mice was decreased after desensitization of CXCR4 with an *in vitro* exposure to CXCL12 but also after pre-treatment with pertussis toxin—inhibitor of the chemokine receptor Gαi-mediated signaling—or with the CXCR4 inhibitor AMD3100 (62, 66). As expected, Nalm6 cells injected to immunodeficient mice home to BM peri-sinusoidal regions in the vicinity of the CXCL12-expressing PSS cells (66). Importantly, in childhood B-ALL, higher levels of CXCR4 were correlated to an increased extramedullary organ infiltration and to a poor outcome. However, it is not clear whether high CXCR4 is a prognosis factor for extramedullary relapse (67, 68). In adult, overall survival was correlated to phosphorylated CXCR4 but not to CXCR4 expression, indicating that activated CXCR4 may be a better prognosis factor (69). Larger cohorts will be needed to confirm these results.

The pre-BCR is expressed by a B-ALL subset equivalent to pre-B cells which is identified as being intracellular Igµ positive (icµ^+^) (70). Importantly, phosphorylation of key tyrosine kinases downstream of the pre-BCR in icµ^+^ patient samples is increased and treatment with an anti IgH antibody effectively stimulates pre-BCR signaling (71). In addition, similarly to normal mouse pre-B cells, co-culture of the human pre-BCR^+^ cell line Nalm6 with stromal cells induced a GAL1-dependent clustering of the pre-BCR at the leukemic/stromal cell synapse and activation of the pre-BCR (37–39). Stromal cells are therefore likely to give a proliferative advantage to pre-BCR^+^ leukemic cells through direct contacts and production of GAL1.

In addition to stromal cells, adipocytes were also shown to play protective roles in B-ALL. CXCL12 produced by adipocytes induces the migration of leukemic cells into adipose tissue (72, 73). Interestingly, co-cultures of B-ALL cell lines with the 3T3-L1 cell line differentiated in adipocytes or with adipose tissue induce a resistance to chemotherapeutic treatments (72, 74). Furthermore, while the overall survival of obese C57Bl/6 mice transplanted with the murine leukemic 8093 cell line was similar to control mice, their survival upon Vincristine treatment was decreased (74). The 8083 cell line treated *in vitro* with Vincristine was found to upregulate the anti-apoptotic proteins Pim2 and Bcl2 when co-cultured in presence of the 3T3-L1 fibroblasts differentiated in adipocytes as compared to undifferentiated cells. It has been further demonstrated in the case of human T-ALL transplanted to immunodeficient mice, that leukemic cells are more quiescent and metabolically less active in the BM of adipocyte-rich tail vertebrae or in gonadal adipose tissue than in the BM of adipocyte-poor femur or thoracic vertebrae (75). Adipocyte-dependent molecular cues controlling leukemic cell survival and quiescence remain to be identified. Although these different results point to a role of adipose tissue and adipocytes in the resistance to treatments, the correlation between obesity and outcome remains controversial and needs to be confirmed through the analysis of large cohorts of patients (76).

Finally, the cross-talk between endothelial cells and B-ALL has been poorly studied. B-ALL patients show an increased microvascularization in the BM associated to an increase in most of the cases of the pro-angiogenic factor basic fibroblast growth factor (bFGF) but not of vascular endothelial growth factor (VEGF) (77, 78). The importance of angiogenesis in B-ALL prognosis is still controversial and has been described in details elsewhere (79). It has been however shown *in vitro* that endothelial cells protect B-ALL blasts from apoptosis through over-expression of the anti-apoptotic protein Bcl-2 (78). Further investigations will be needed to confirm this result and identify the molecular interactions taking place. Vascular remodeling is a feature of leukemia progression. It has been shown in a murine model of BCR-ABL1 B-ALL that during leukemia progression, there is an increase in blood vessel density and therefore a higher oxygen supply (80). However, because of high O_2_ consumption by leukemic blasts and despite an important extracellular O_2_ supply, intracellular hypoxia is elevated. In advanced disease stages, the O_2_ supply becomes limited and the extracellular space highly hypoxic. As a consequence of hypoxia, HIF-1α was found to be stabilized in B-ALL and high HIF-1α levels were related to a poor outcome and resistance to chemotherapy through a decrease in pro-apoptotic and an increase in anti-apoptotic proteins (81, 82). Interestingly, co-culture of B-ALL cells with the MS-5 mesenchymal cell line further enhanced HIF-1α protein levels through AKT phosphorylation in hypoxic conditions, and through ERK phosphorylation independently of hypoxia (81).

## Immune Cells in the Leukemic Niche

Tumor progression relies on the capacity of malignant cells to evade the immune system. In the BM, immune protection is supported by regulatory T cells (Tregs), which were shown to protect allogeneic HSC from rejection (83). Furthermore, Treg depletion in mouse leads to a differentiation arrest at the pre-proB stage (84). Tregs were found to control B cell differentiation through the regulation of IL7 production by PSS cells. Therefore, BM can be considered as an immune-privileged site. In the case of B-ALL, the proportion of CD4^+^CD25^+^ Tregs was found to be slightly but significantly increased for B-ALL patients at diagnosis in two independent studies (85, 86). Furthermore, Tregs from B-ALL (and T-ALL) patients secreted lower levels of the T cell stimulating cytokine IL-2 *in vitro*, and higher levels of the immunosuppressive IL-10 and TGFβ, as compared to healthy individuals (85). In contradiction with these results, another study showed a decrease in CD4^+^CD25^+^ Tregs (87). However, by refining the immunophenotyping the authors found a strong increase in the proportion of functional FoxP3^+^ and IL-10^+^ Tregs. Furthermore, Tregs from B-ALL patients secreted higher levels of IL-10 and TGFβ, and inhibited T cell proliferation *in vitro* more strongly than Tregs from healthy individuals. Altogether these results suggest that B-ALL may escape immune-surveillance by activating Tregs.

Natural Killer (NK) cells are cells of the innate immune system which have the capacity to kill their target cell and are involved in tumor immune surveillance (88). Accordingly, B-ALL patients with a higher proportion of NK cells in the BM respond better to treatment (89). However, the frequency of CD56^+^CD3^-^ NK cells is decreased for most patients as compared to healthy individuals (90, 91). Interestingly, expression of the activating receptor NKp46 was decreased while the inhibitory receptor NKG2A was increased (91). Furthermore, NK cells from patients had a decreased capacity to degranulate, to secrete IFNγ and to induce the lysis of target cells compared to NK cells from healthy controls. Leukemic cells were further shown to directly inhibit NK cell cytotoxic activity and thus to drive immune evasion partly through the secretion of TGFβ and the activation of the Smad2/3 signaling pathway in NK cells.

B-ALL also has the capacity to evade the immune system by inhibiting macrophage phagocytosis. CD47, an inhibitor of phagocytosis by macrophages (92), is overexpressed by a subset of B-ALL, and was found to be an indicator of poor survival and associated with a high risk of refractory disease (93). Furthermore, it has been demonstrated in a humanized mouse model of leukemia/lymphoma that leukemic cells expressing a shRNA specific for the prostaglandin synthetase 3 gene (PTGES3) were more sensitive to antibody-dependent cell-mediated cytotoxicity (ADCC) (94). This result is in agreement with the fact that PTGES3 is involved in the catalysis of prostaglandin E2, an inhibitor of macrophage phagocytosis (95).

Finally, a recent study performed an in depth analysis of the immune microenvironment of B-ALL patient samples by single cell RNAseq (96). Although the myeloid compartment was strongly diminished in the BM of B-ALL patients at diagnosis compared to healthy individuals, the frequency of CD14^+^CD16^+^ non-classical monocytes was increased at the expense of CD14^+^CD16^-^ classical monocytes. Importantly, the frequency of this population strongly decreased upon remission but re-emerged at relapse. Furthermore, the authors found that a high monocyte count in both adult and childhood B-ALL was associated with a lower overall survival (OS) and relapse free survival (RFS). Finally, in a murine model of BCR-ABL1^+^ B-ALL, mice transplanted with leukemic cells were treated with an antibody specific for CSF1R, highly expressed by non-classical monocytes (antibody AFS98), with Nilotinib, a BCR-ABL1 tyrosine kinase inhibitor (TKI) or a combination of both. The antibody-induced depletion of the non-classical monocyte subset had no effect on survival, however, the antibody improved the responsiveness of mice to Nilotinib. This result suggests that the non-classical monocyte subpopulation may promote leukemic blast survival and protect them from treatment.

## Treatments Targeting the Leukemic Niche

Novel therapeutics, have been introduced in the standard treatment regimens based on multiagent chemotherapies. TKIs which target dysregulated signaling pathways have given promising results with improved overall survival (97). Immune cells can also be addressed to leukemic cells through the use of monoclonal antibodies targeting surface receptors. As an example, Blinatumomab is a CD3-CD19 bispecific T-cell engaging antibody able to activate T-cells without the need for additional costimulatory signals. Blinatumomab is FDA approved for the treatment of MRD^+^ or relapsed/refractory B-ALL (98, 99). However, part of the patients does not respond to Blinatumomab treatment. Exhaustion markers including PD-1 and CTLA-4 were upregulated by T cells following treatment, together with an upregulation of their respective ligands PDL-1 and CD86 at the surface of leukemic cells (100, 101). The *in vitro* Blinatumomab-mediated T cell response was inhibited when patient T cells as compared to healthy donor T cells were incubated with ALL cells. However, this block was relieved by using an anti-PD-1 alone or together with an anti CTLA-4. Blinatumomab non responders were also found to have an increased proportion of Tregs which impair T cell response in a contact-dependent manner (102). Clinical trials are currently underway to determine the relevance of combining Blinatumomab with checkpoint inhibitors like the anti PD-1 Pembrolizumab (103).

Immunotherapies have moved a step forward with the advance on chimeric antigen receptor (CAR) T‐cell treatments. CAR-T cells are amplified from autologous T cells from the patient. They are engineered to express a single‐chain variable fragment (scFv) of an antibody specific for a tumor cell marker (e.g. anti CD19 antibody), fused to the CD3ζ stimulatory domain and to an additional costimulatory domain in the case of second-generation therapies. CAR-T cells have been approved in 2017 by the FDA for the management of refractory or second/later relapsed ALL (104).

Mechanisms involved in the evasion from innate immune response can also be targeted. CD47, shown to be overexpressed by B-ALL, inhibits phagocytosis by macrophages. Treatment with the anti CD47 neutralizing antibody B6H12.2 relieved the block on phagocytosis *in vitro* and impaired leukemia engraftment *in vivo* (**Figure 2**) (93). In a humanized mouse model of leukemia, resistance to the anti CD52 antibody Alemtuzumab used to target CD52^+^ lymphoid leukemia by ADCC was observed in the BM but not in periphery (94). This resistance was linked to a decrease in macrophage numbers at the time of BM invasion by leukemic cells. Importantly, most mice treated with a combination of Alemtuzumab and the alkylating agent cyclophosphamide (CTX) survived for at least 6 months while all mice under monotherapies died in about 2 months. CTX was found to specifically induce production by the BM microenvironment of TNFα, VEGF, and CCL4, at the origin of an increased penetrance of macrophages in the BM as well as the activation of their phagocytic properties. Altogether these results clearly demonstrated that reactivation of the immune microenvironment of the BM can improve the therapeutic outcome.

As leukemic niches have the capacity to give pro-survival and quiescence signals through direct interactions, it has been postulated that targeting the crosstalk between non immune cells of the BM microenvironment and leukemic cells could improve current therapies. A high expression of VLA-4 in a cohort of MRD^+^ patient is of bad prognosis (58). In a PDX model of B-ALL, the use of Natalizumab, an anti-VLA-4 monoclonal antibody, in combination with a multi-agent chemotherapy led to a complete remission of all animals for at least 4 months after treatment, while mice with the chemotherapy alone died less than 2 months after treatment (58). The anti VLA-4 alone marginally or did not improve survival (57, 58) therefore supporting that the loss of adhesion of leukemic cells with their niche sensitizes them to chemotherapy.

CXCR4 inhibitors have also been tested in B-ALL. The T140 and AMD3100 (Plerixafor) inhibitors impaired *in vitro* migration of the Nalm6 cell line and patient samples towards a CXCL12 gradient and into stromal layers (105). Furthermore, the loss of contact with stromal cells induced by the inhibitors increased the proliferative status of leukemic cells and their sensitivity to chemotherapeutic drugs. Treatment of mice engrafted with B-ALL from patients with plerixafor induced mobilization of leukemic cells in the circulation with a decreased tumor burden in the spleen but not in the BM (106, 107). Nevertheless, treatment of PDX mice with a combination of Plerixafor and the antimetabolite Ara-C led to a significant decrease in the tumor burden of the spleen as well as the BM compared to the Ara-C monotherapy (107).

Finally, pre-BCR signaling has been shown to be effective in cµ^+^ B-ALL and to rely on contacts with stromal cells (37, 39, 71). As a consequence, *in vitro* treatment of patient samples with TKIs specific for kinases involved in pre-BCR signaling (Ibrutinib and Dasatinib: BTK inhibitors; PRT062607: Syk inhibitor) induces a strong death at low half maximal inhibitory concentration (IC50) (71). Furthermore, survival of mice xenografted with pre-BCR^+^ B-ALL is increased upon treatment with Ibrutinib or Dasatinib, and a synergistic effect of Ibrutinib was observed *in vitro* in combination with either Dexamethasone or Vincristine (71, 108).

## Conclusion

B-ALL mutational status has been extensively studied; however, the crosstalk of B-ALL with their immune and non-immune microenvironment remains poorly characterized despite their known function in the control of residual disease and relapse. Chemotherapy toxicity and secondary effects are also a heavy burden for survivors, and there would be a strong benefit to develop adjuvant targeted therapies. Tremendous progress has been made in the outcome of patients by reactivating the immune tumor microenvironment through the development of immunotherapies. Nevertheless resistance and relapse in B-ALL still concerns an important number of patients, particularly among elderly. One of the main challenges remains to understand how the interactions between partner cells are orchestrated into the leukemic microenvironment in order to find which of these interactions could be inhibited to lead to the collapse of the leukemic cell ecosystem. Mouse models have been essential in understanding the organization of the BM non-immune microenvironment and its importance in normal and pathological hematopoietic progenitor maintenance and development. Thanks to advances in single cell transcriptomics and high resolution microscopy, the spatial organization of the BM starts to be resolved (109). However, the translation to human BM organization is more difficult due to the paucity of BM samples. PDX models are valuable for the analysis of the non-immune microenvironment, but the simultaneous contribution of immune cells cannot be evaluated. In addition, one has to consider that B-ALL are composed of different subtypes with diverse mutational landscapes. The composition of their supportive microenvironment and their dependence towards it may therefore be strongly dependent on their intrinsic properties. Therefore, future studies may consist in the establishment of experimental immunocompetent models in which new adjuvant therapies may be tested. This will require to better understand the natural history of microenvironment shaping during leukemic development starting from LIC in order to identify key steps in B-ALL ecosystem establishment. No doubts that mathematical modeling and new technological breakthroughs such as single cell technology will lead to the identification of cellular cross-talks constituting the Achilles heel for the development of efficient innovative therapies.

## Author Contributions

SM and MA-L conceptualized and finalized the manuscript. MD and K-IS provided the content and prepared the figures. JP provided the content. All authors contributed to the article and approved the submitted version.

## Funding

This work was supported by grants from the Ligue Nationale contre le Cancer (EL2020). MD is supported by the Ligue Nationale Contre le Cancer (IP/SC-16060) and K-IS by the Algerian “Ministère de l’enseignement supérieur et de la recherche scientifique” (MESRS). JP was the recipient of a conjoint PhD grant from the Région PACA/Inserm/Innate Pharma and from la Ligue Nationale contre le Cancer (#TDCU19117).

## Conflict of Interest

The authors declare that the research was conducted in the absence of any commercial or financial relationships that could be construed as a potential conflict of interest.
